# Laboratory and Clinical Predictors of Disease Progression following Initiation of Combination Therapy in HIV-Infected Adults in Thailand

**DOI:** 10.1371/journal.pone.0043375

**Published:** 2012-08-15

**Authors:** Trinh Duong, Gonzague Jourdain, Nicole Ngo-Giang-Huong, Sophie Le Cœur, Pacharee Kantipong, Sudanee Buranabanjasatean, Prattana Leenasirimakul, Sriprapar Ariyadej, Somboon Tansuphasawasdikul, Suchart Thongpaen, Marc Lallemant, for the Program for HIV Prevention and Treatment (PHPT) Study Group

**Affiliations:** 1 Institut de Recherche pour le Développement (IRD UMI 174), Paris, France; 2 MRC Tropical Epidemiology Group, London School of Hygiene and Tropical Medicine, London, United Kingdom; 3 Faculty of Associated Medical Sciences, Chiang Mai University, Chiang Mai, Thailand; 4 Harvard School of Public Health, Boston, Massachusetts, United States of America; 5 Institut de Recherche pour le Développement, UMR 196 CEPED, Université Paris Descartes INED- IRD, Paris, France; 6 Chiang Rai Prachanukroh Hospital, Chiang Rai, Thailand; 7 Mae Chan Hospital, Chiang Rai, Thailand; 8 Nakornping Hospital, Chiang Mai, Thailand; 9 Rayong Hospital, Rayong, Thailand; 10 Buddhachinaraj Hospital, Pitsanuloke, Thailand; 11 Mahasarakam Hospital, Mahasarakam, Thailand; IPO, Inst Port Oncology, Portugal

## Abstract

**Background:**

Data on determinants of long-term disease progression in HIV-infected patients on antiretroviral therapy (ART) are limited in low and middle-income settings.

**Methods:**

Effects of current CD4 count, viral load and haemoglobin and diagnosis of AIDS-defining events (ADEs) after start of combination ART (cART) on death and new ADEs were assessed using Poisson regression, in patient aged ≥18 years within a multi-centre cohort in Thailand.

**Results:**

Among 1,572 patients, median follow-up from cART initiation was 4.4 (IQR 3.6–6.3) years. The analysis of death was based on 60 events during 6,573 person-years; 30/50 (60%) deaths with underlying cause ascertained were attributable to infections. Analysis of new ADE included 192 events during 5,865 person-years; TB and *Pneumocystis jiroveci* pneumonia were the most commonly presented first new ADE (35% and 20% of cases, respectively). In multivariable analyses, low current CD4 count after starting cART was the strongest predictor of death and of new ADE. Even at CD4 above 200 cells/mm^3^, survival improved steadily with CD4, with mortality rare at ≥500 cells/mm^3^ (rate 1.1 per 1,000 person-years). Haemoglobin had a strong independent effect, while viral load was weakly predictive with poorer prognosis only observed at ≥100,000 copies/ml. Mortality risk increased following diagnosis of ADEs during cART. The decline in mortality rate with duration on cART (from 21.3 per 1,000 person-years within first 6 months to 4.7 per 1,000 person-years at ≥36 months) was accounted for by current CD4 count.

**Conclusions:**

Patients with low CD4 count or haemoglobin require more intensive diagnostic and treatment of underlying causes. Maintaining CD4≥500 cells/mm^3^ minimizes mortality. However, patient monitoring could potentially be relaxed at high CD4 count if resources are limited. Optimal ART monitoring strategies in low-income settings remain a research priority. Better understanding of the aetiology of anaemia in patients on ART could guide prevention and treatment.

## Introduction

By the end of 2009, 5 of the 33 million HIV-infected patients in low- and middle-income countries were receiving antiretroviral therapy (ART) [1]. Minimizing long-term morbidity and mortality in patients on ART becomes increasingly important as treatment programmes mature, standard of care improves and more effective drug combinations are available. This has led to increased debate on optimal approaches for monitoring antiretroviral treatment in low-income settings [2]. Accurate data on determinants of long-term disease progression in treated patients may therefore inform patient management guidelines and research directions.

Differences between low and high-income settings such as patients’ characteristics at presentation to HIV medical services, prevalence of co-morbidities, distribution of AIDS-defining illnesses, spectrum of causes of death, and clinical management have generally been observed [3]. It is therefore important to assess not only key predictors of long-term outcomes in patients on ART for different settings, but also the nature of these associations.

So far, analyses from low and middle-income settings on prognostic factors in treated patients have mostly considered baseline characteristics only and/or included relatively short-term follow-up [3], [4], [5], [6], [7], [8], [9], [10], [11]. Only a few have assessed effects of CD4 count and other laboratory markers while on ART on long-term outcomes [12], [13], [14], [15], [16]. Consistent with data from Europe and North America [17], [18], [19], [20], [21], these showed current CD4 cell count was the strongest predictor of mortality [13], [14], [16] and current haemoglobin independently predictive [13], [16]. The effect of current viral load was not consistently observed across studies [13], [14], [16]. However, some of these studies from low-income settings had certain limitations, including: the effect of current CD4 count was estimated without adjusting for current viral load and other markers [12], [15]; CD4 count was categorised in analysis with levels above 200 cells/mm^3^ combined, restricting scope to identify whether there is a target threshold above which CD4 level should be maintained to minimise mortality [12], [16]; and information on cause of death was not reported, limiting interpretation [13], [14], [15].

Using data from a multi-centre cohort in Thailand, a middle-income country, we assessed the prognostic effects of CD4 count, viral load and haemoglobin at start of combination antiretroviral therapy (cART) and after treatment initiation on progression to death and to first new ADE. The effect of new ADE on death was also examined.

## Methods

### Ethics Statement

The Program for HIV Prevention and Treatment (PHPT) cohort was approved by the Thai Ministry of Public Health and local ethics committees. Participants provided written informed consent at entry.

### Study Background

The PHPT adult cohort prospectively followed HIV-infected patients receiving ART in 40 public hospitals across Thailand (NCT00433030 www.clinicaltrials.gov). The study has been previously described [22]. Briefly, the cohort began in 1999, recruiting women from trials on prevention of mother-to-child transmission of HIV (PMTCT) [23], [24], and later extended to partners of these women and any HIV-infected adults presenting at participating sites. The criteria for initiation of therapy was CDC clinical stage B/C or CD4<250 cells/mm^3^ [25]. Initial HAART regimens changed over time with increased availability of drugs. Alternative drugs were available in case of intolerance or confirmed virologic failure. Cotrimoxazole and fluconazole prophylaxis were prescribed as needed. Patients attended the clinic monthly for physical examination, drug refills and adherence counselling conducted by a nurse. They were also reviewed by a physician every month during the first 3 months of treatment and at 3-monthly intervals thereafter, with additional referrals as required. CD4 and virology testing and a complete blood cell count were done at start of treatment, at 3 months and every 6 months thereafter. Cause of death was reported by site physicians, and further reviewed and classified by two independent physicians based on ICD-10 classification (http://www.who.int/classifications/icd/en/index.html).

### Statistical Analyses

Patients were included in these analyses if they were ART naïve at PHPT cohort enrolment (apart from treatment received for PMTCT), subsequently started cART with at least 3 antiretroviral drugs at age 18 years or older, and had at least one CD4 and one viral load evaluation after cART initiation.

Outcomes evaluated were progression from cART initiation to (i) all-cause mortality and (ii) first new ADE (which included death in the absence of a new AIDS diagnosis after starting cART). In the analysis of new ADE, patients with AIDS diagnosis before starting cART were included and progression to the first clinically new ADE was considered, ignoring recurrence of ADE’s occurring before cART initiation. Follow-up was considered from date starting cART up to the date of the outcome of interest or date of last clinic visit for patients censored.

At a given time point, we defined current CD4 count, viral load or haemoglobin as the most recent measurement taken after cART initiation which was within the last 9 months of that time point. The value at cART initiation was defined as the most recent measurement within 6 months prior to date of initiation. We classified ADEs as either mild or moderate/severe, based on prognostic categories proposed in a Antiretroviral Therapy Cohort Collaboration (ART-CC) study which showed different types of ADE diagnosed after cART initiation had varying impact on mortality [26].

First, we examined the predictive value of the following time-varying factors: current CD4 count; current viral load; current haemoglobin; time since cART initiation; and for progression to death only, diagnosis of new mild ADEs and of new moderate/severe ADEs after cART initiation. The effect of type of new ADEs on mortality was assessed based on patients without prior AIDS diagnosis at cART initiation, consistent with the ART-CC study [26]. Time-dependent indicator variables corresponding to before and after diagnosis of the first event (s) within each ADE prognostic category were fitted.

We then assessed whether after accounting for current CD4 count, viral load and haemoglobin, there were any additional prognostic value in CD4 count, viral load, haemoglobin and AIDS diagnosis status at cART initiation.

**Table 1:** Patients’ characteristics at cART initiation (N = 1572).

	Number of patients	(%)^a^
Female sex	1192	(76%)
HBV antigen positive	93	(6%)
Anti-HCV seropositive	49	(4%)
Previous ART treatment		
None (except for PMTCT ART use)	1462	(93%)
Dual therapy	110	(7%)
Age (years)		
median (IQR)	32.7	(28.5–38.1)
<30	541	(34%)
30–39	736	(47%)
40–49	243	(15%)
≥50	52	(3%)
Year started cART		
2001–2002	270	(17%)
2003–2004	406	(26%)
2005–2006	766	(49%)
2007–2010	130	(8%)
CD4 cell count (cells/mm^3^)		
median (IQR)	129	(59–198)
<50	324	(21%)
50–99	299	(17%)
100–149	258	(17%)
150–199	282	(18%)
200–349	342	(22%)
≥350	35	(2%)
Viral load (log copies/ml), median (IQR)	4.8	(4.3–5.2)
Haemoglobin (g/dl)		
median (IQR)	11.7	(10.6–2.8)
<8	18	(1%)
8–9.99	199	(13%)
10–11.99	627	(41%)
≥12	669	(44%)
AIDS diagnosis before cART initiation	344	(22%)
No	1224	(78%)
Previously diagnosed with mild ADE (s) only^b^	247	(16%)
Previously diagnosed with moderate/severe ADE (s)^b^	101	(6%)
Initial cART regimen		
Efavirenz+ NRTI’s	584	(37%)
Nevirapine + NRTI’s	199	(13%)
PI + NRTI’s	743	(47%)
NNRTI + PI + NRTI’s	46^c^	(3%)

cART, combination antiretroviral therapy; HBV, hepatitis B virus; HCV, hepatitis C virus; PMTCT, prevent of mother-to-child transmission; ADE, AIDS-defining event; NNRTI, non-nucleoside reverse transcriptase inhibitor; NRTI, nucleoside reverse transcriptase inhibitor; PI, protease inhibitor; IQR, inter-quartile range.

a Percentage based on total with data available. Number with missing data: sex (1), HCV status (269), CD4 count (32), HIV-1 RNA (137), haemoglobin (59).

b ADEs were defined as either mild or moderate/severe based on prognostic categories proposed by Mocroft et al [26]. Note only 2 patients were previously diagnosed with a severe ADE.

c The 46 patients starting with regimens containing NNRTI+PI were all previously on dual therapy.

Effects of covariates were estimated using Poisson regression models, based on follow-up periods with both current CD4 count and viral load available. Clustering within hospitals was included as a random effect. Multivariable analyses were adjusted for time since cART initiation (split according to <6 months, 6–11.99, 12–23.99, 24–35.99 and ≥36 months), current CD4 count, viral load and haemoglobin and, in addition, the following *a priori* confounders at initiation which were selected on the basis of findings from previous studies [7], [22], [27], [28]: sex, HBV and HCV status, CD4 count, viral load, haemoglobin, age, calendar period (before 2005, 2005 onwards), AIDS diagnosis status, and whether ART-naïve when starting cART.

CD4 count was square root transformed to improve model fit. Current viral load was categorized as <400, 400 to 99,999 and ≥100,000 copies/ml; these categories were chosen since the detection limit of assays used had varied over time and a previous study showed high viral load at 6 months from starting treatment was associated with poorer survival but only at levels above 100,000 copies/ml [29]. Viral load and age at cART initiation were log transformed. Non-linear effects were assessed for using cubic spline terms with knots at the 10^th^, 50^th^ and 90^th^ centiles [30]. Non-proportional hazards were assessed by testing for interaction between covariates and follow-up time (categorised as less or greater than 2 years from cART initiation). Missing data for covariates at cART initiation were imputed using Multivariate Imputation by Chained Equations based on 20 cycles [31].

Finally, we carried out sensitivity analyses with: (i) current CD4 count, viral load and haemoglobin defined assuming a given measurement was valid for a maximum of 6 and 12 months, rather than 9 months and (ii) including only patients who were ART-naïve at cART initiation (apart from ART use for PMTCT).

All statistical analyses were undertaken in STATA, version 11.

**Figure 1:**
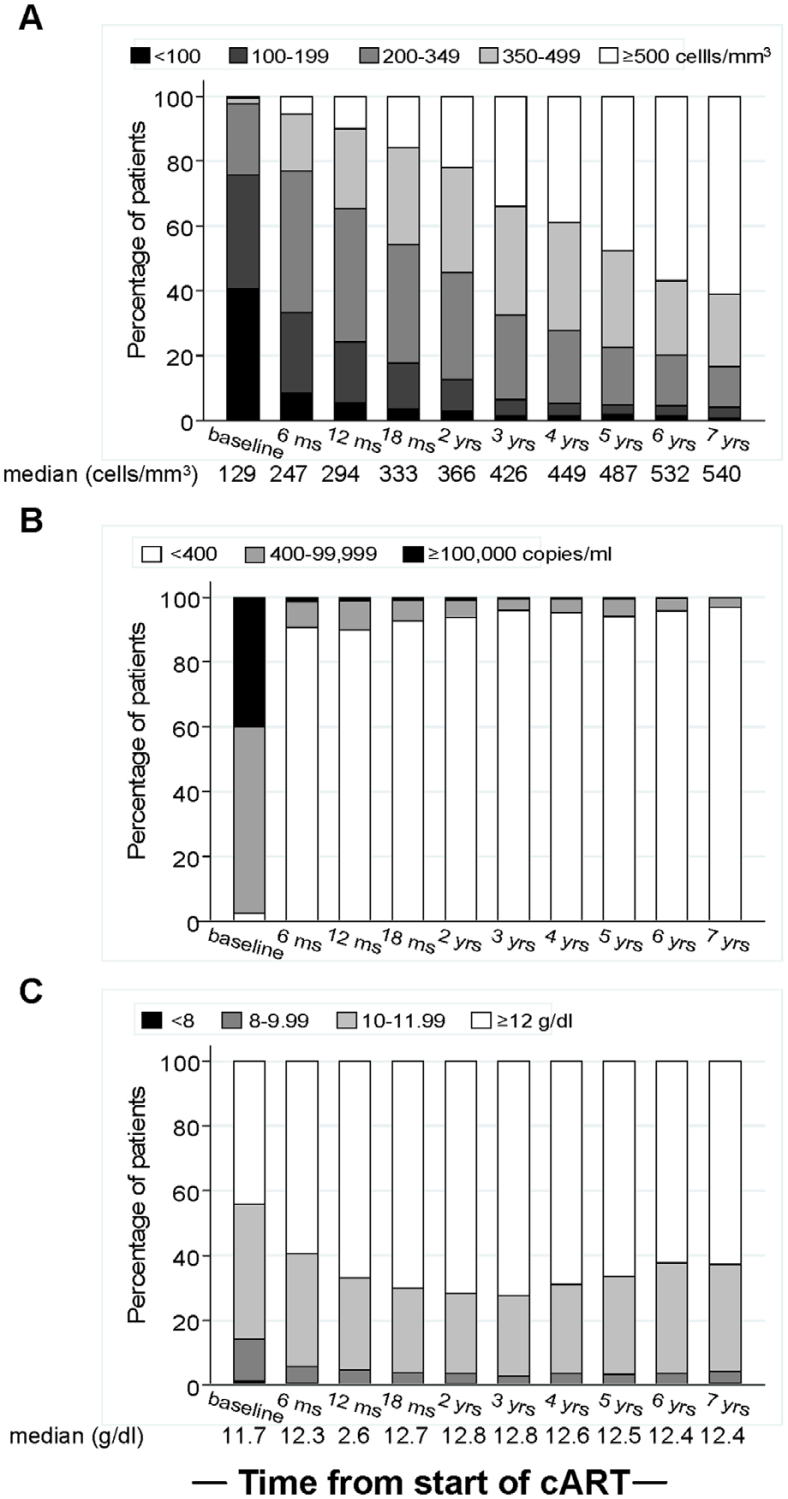
Distribution of CD4 count (A), viral load (B) and haemoglobin (C) over time from initiation of cART. The closest measurement to each nominal time points within a±3 months window was selected for each patient.

## Results

1740 patients were ART-naïve at PHPT cohort enrolment and subsequently started cART aged ≥18 years between 2001 and 2010. Of these, 1572 (90%) had follow-up period (s) after initiation with both current CD4 count and viral load measures and were included in analyses.

### Characteristics at cART Initiation

Three-quarters (76%) of patients were female; Table 1. At cART initiation, median age was 32.7 (IQR 28.5–38.1) years and CD4 count 129 (59–198) cells/mm^3^. Women had lower haemoglobin than men, with median 11.5 (IQR 10.5–12.4) versus 12.8 (11.4–14.2), respectively. A small proportion of patients (7%, n = 110) previously received ART for their HIV, all initially prescribed didanosine plus stavudine dual therapy at median 2.9 (IQR 2.2–3.3) years before switching to cART. Around a fifth of patients (22%, n = 344) were diagnosed with AIDS before cART initiation.

### Follow-up after cART Initiation

Patients were followed up for a median 4.4 (IQR 3.6–6.3) years after starting cART. 145/1572 (9%) patients were lost to follow-up after cART initiation and 149 (9%) voluntarily withdrew. Median number of measurements per patient after initiation was 14 (IQR 11–17) for CD4 count, 14 (10–16) for viral load and 20 (16–27) for haemoglobin.

**Table 2:** Number of patients, events and years of follow-up contributing to analyses.

	Analysis of progression to death	Analysis of progression to first new ADE
Number of patients	1572	1503^a^
Total follow-up (years)	6573	5865
Total number of events	60	192^b^
Time from cART initiation to event (months)		
<6	5 (8%)	27 (14%)
6–11.99	12 (20%)	29 (15%)
12–23.99	21 (35%)	65 (34%)
24–35.99	9 (15%)	32 (17%)
≥36	13 (22%)	39 (20%)

ADE, AIDS-defining event; cART, combination antiretroviral therapy.

a 69 of the 1572 patients included in analyses of progression to death developed a new ADE after cART initiation but before having at least 1 CD4 count and viral load evaluation while on treatment, and therefore were excluded in the analyses of progression to first new ADE.

b 153 patients progressed to ≥1 new ADE (s), while 39 patients died without having a new ADE after starting cART.

**Figure 2:**
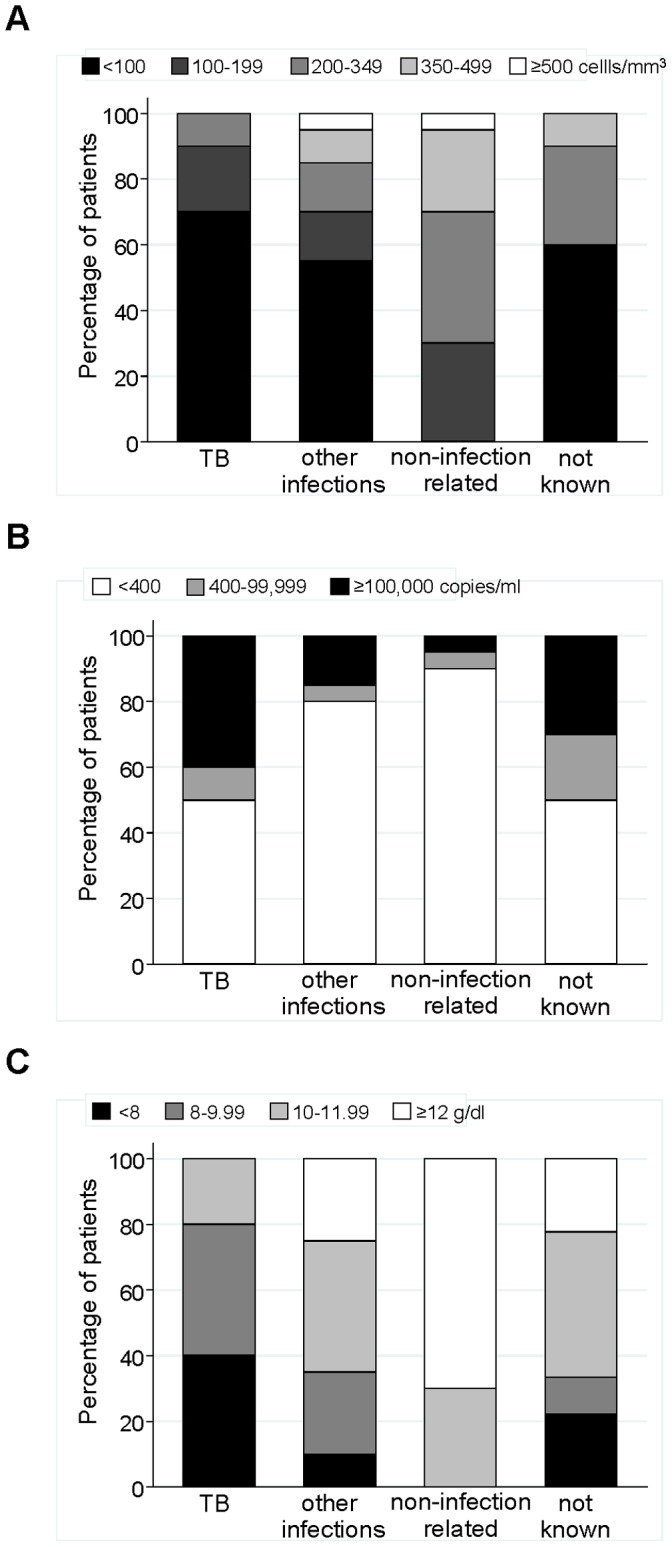
Distribution of most recent CD4 count (A), viral load (B) and haemoglobin (C) measurement at time of death, by cause of death. Based measurements within the last 9 months before date of death. Number of cases by cause of death: 10 TB, 20 other infections, 20 not related to infection and 10 not known.

### Trends in CD4 Cell Count, Viral Load and Haemoglobin after cART Initiation

The proportion of patients with HIV-1 RNA <400 copies/ml was 91.3% at 6 months from cART initiation, and around 95% from 3 years onwards (Figure 1). At 12 months, 11% of patients with viral load suppression <400 copies/ml attained CD4 level ≥500 cells/mm^3^, increasing to 35% and 52% at 3 and 5 years, respectively. High CD4 count at cART initiation was associated with high absolute level at all subsequent times (data not shown). Haemoglobin increased following cART though mainly within the first year, with lower levels observed in women throughout follow-up (data not shown).

### Follow-up and Events Contributing to Analyses of Disease Progression

The analysis of progression to death was based on 60 deaths during 6573 person-years (90% of total follow-up from cART initiation) when both current CD4 count and viral load were available; Table 2. Cause of death was ascertained in 50/60 (83%) of cases. Of these, 30 (60%) died of infection-related causes (10 tuberculosis, 7 cryptomeningitis, 4 pneumonia, 1 toxoplasmosis, 1 meningitis, 2 septicaemia, 1 cellulitis, 1 disseminated/generalized fungal infection, 1 complication related to hepatitis C co-infection, 1 human papillomavirus infection and 1 cholecystitis) and 20 (40%) of other causes (9 cardiovascular disease, 3 cancer, 1 liver failure, 1 asthma, 4 suicide, 1 alcohol abuse and 1 accident). Among deaths with underlying cause ascertained, those occurring within the first 2 years of cART were more likely to be due to infection-related causes (24/34, 71%) compared with after (6/16, 38%) (p = 0.03). Patients with infection-related causes of death had substantially lower CD4 count and haemoglobin and were less likely to be suppressed <400 copies/ml at the time of death compared with those dying of other known causes; Figure 2.

In the analysis of progression to first new ADE, 69 of the 1572 patients developed a new ADE after cART initiation but prior to any CD4 and viral load evaluation while on treatment, so were excluded; of these, 80% (n = 55) developed a new ADE within 6 months, 46% (n = 32) within 3 months. Among the remaining 1503 patients included, there were 192 events during 5865 person-years (Table 2); 153 patients developed ≥1 new ADE (s) and 39 died without developing a new ADE. Eleven (7%) of the 153 patients developing new ADE (s) presented simultaneously with two or more ADEs at initial diagnosis. The most common first new ADE were: TB (diagnosed in 35% of the 153 patients), *Pneumocystis jiroveci* pneumonia (20%), generalized herpes simplex infection (10%) and recurrent pneumonia (5%).

### Associations with Progression to Death

The associations with mortality for factors after cART initiation are shown in Table 3. Mortality rate was 124.8 per 1000 person-years at CD4 count <100 cells/mm^3^, decreasing substantially to 1.1 per 1000 person-years at ≥500 cells/mm^3^. In multivariable analyses, low level of current CD4 count (p<0.001) and haemoglobin (p<0.001) were the strongest predictors of mortality. Current viral load had a weak independent effect (p = 0.05), with poorer survival associated with levels ≥100,000 copies/ml. As expected, mortality rate declined steadily with time from cART initiation, from 21.3 per 1000 person-years during the first 6 months to 4.7 per 1000 person-years at ≥36 months; however, this trend disappeared after adjusting for current CD4 count (p = 0.82), but not when only either current viral load (p = 0.01) or haemoglobin (p = 0.006) were accounted for.

**Table 3:** Predictive effects of factors after cART initiation on progression to death and to new ADE.

Factors	Progression to death			Progression to first new ADE		
	Rate per 1000 py (events/py)	Adjusted rate ratio^a^ (95% CI)	p-value	Rate per 1000 py (events/py)	Adjusted rate ratio^a^ (95% CI)	p-value
Current CD4 count (cells/mm^3^)						
<100	124.8 (24/192)	33.6 (10.6–106.5)^b^	<0.001	290.7 (44/151)	4.6 (2.5–8.4)^b^	<0.001
100–199	16.0 (11/686)	12.8 (5.6–29.7)		59.9 (36/601)	2.6 (1.6–4.1)	
200–349	7.7 (15/1951)	5.4 (3.1–9.3)		34.5 (61/1769)	1.7 (1.1–2.6)	
350–499	4.2 (8/1917)	2.5 (1.9–3.4)		18.3 (32/1753)	1.1 (0.8–1.6)	
≥500	1.1 (2/1826)	1		11.8 (19/1613)	1	
Current viral load (copies/ml)						
<400	7.1 (44/6180)	1	0.05	27.4 (152/5546)	1	0.03
400–99,999	14.6 (5/343)	0.5 (0.2–1.5)		73.5 (22/299)	1.3 (0.8–2.2)	
≥100,000	218.9 (11/50)	2.1 (0.8–5.6)		429.8 (18/42)	2.4 (1.2–4.8)	
Current haemoglobin (g/dl)						
<8	419.4 (8/19)	12.0 (5.7–25.1)^b^	<0.001	530.8 (9/17)	4.1 (2.3–7.1)^b^	<0.001
8–9.99	43.0 (10/233)	4.4 (2.8–6.8)		122.2 (24/196)	2.3 (1.7–3.2)	
10–11.99	10.7 (20/1877)	1.8 (1.4–2.2)		34.1 (58/1701)	1.4 (1.2–1.6)	
≥12	4.8 (21/4417)	1		23.8 (94/3955)	1	
Diagnosis of new mild ADEs after cART initiation^c^						
Before diagnosis	5.9 (29/4900)	1		–		
After initial diagnosis	36.2 (10/276)	4.2 (1.9–9.7)	0.001			
Diagnosis of new moderate/severe ADEs after cART initiation^c^						
Before diagnosis	6.7 (34/5038)	1		–		
After initial diagnosis	36.2 (5/138)	5.3 (1.8–15.8)	0.003			
Time since cART initiation (months)						
<6	21.3 (5/235)	1	0.86	118.4 (27/228)	1	<0.001
6–11.99	16.5 (12/726)	1.0 (0.3–2.9)		42.4 (29/683)	0.3 (0.2–0.6)	
12–23.99	14.5 (21/1445)	1.4 (0.5–3.8)		49.1 (65/1324)	0.5 (0.3–0.9)	
24–35.99	6.5 (9/1376)	1.0 (0.3–3.1)		26.1 (32/1224)	0.4 (0.2–0.7)	
≥36	4.7 (13/2790)	1.0 (0.3–3.1)		16.1 (39/2427)	0.3 (0.2–0.5)	

cART, combination antiretroviral therapy; ADE, AIDS-defining event; py, person-years; CI, confidence interval.

a Effects were estimated adjusted for *a priori* confounders, time since cART initiation, and current CD4 count, viral load and haemoglobin. Current CD4 count and haemoglobin, and CD4 count, viral load and haemoglobin at cART initiation were analysed as continuous variables.

b To present the estimated effect of CD4 count (fitted square root transformed) and current haemoglobin (fitted with additional cubic spline term), we derived from the fitted model the rate at the marker value at which 50% of person-years of follow-up falls above and below within each strata, and then calculated the corresponding rate ratios.

c Based on patients without prior AIDS diagnosis before cART initiation. Only 1 patient was diagnosed with a severe ADE after starting cART, who had progressive multifocal leukoencephalopathy.

Table 4 shows the predictive effects of factors at cART initiation after adjusting for current CD4 count, haemoglobin and viral load. At a given current CD4 count value, lower CD4 count at cART initiation was weakly associated with decreased mortality, indicating better prognosis with greater increase in CD4 count from baseline. Viral load and haemoglobin at cART initiation had no effect.

**Table 4:** Predictive effects of factors at cART initiation on progression to death and to new ADE, adjusted for current CD4 count, haemoglobin and viral load.

Factors	Progression to death			Progression to first new ADE		
	Rate per 1000 py (events/py)	Adjusted rate ratio^a^ (95% CI)	p-value	Rate per 1000 py (events/py)	Adjusted rate ratio^a^ (95% CI)	p-value
CD4 count at initiation (cells/mm^3^)						
<100	12.0 (32/2657)	0.5 (0.3–1.0)	0.07	53.0 (115/2168)	1.4 (0.9–2.1)	0.11
100–199	8.2 (18/2206)	0.8 (0.6–1.0)		26.7 (55/2061)	1.1 (1.0–1.3)	
≥200	5.8 (9/1552)	1		12.5 (19/1515)	1	
Viral load at initiation (copies/ml)		1.3 (0.9–1.9) per log_10_(copies/ml)	0.23		1.6 (1.2–2.0) per log_10_(copies/ml)	<0.001
<10,000	5.9 (6/1011)			12.2 (12/986)		
10,000–99,999	5.5 (14/2561)			25.0 (58/2323)		
≥100,000	13.4 (31/2306)			53.3 (104/1950)		
Haemoglobin at initiation (g/dl)		1.0 (0.9–1.1) per unit	0.93		1.0 (0.9–1.0) per unit	0.41
<8	12.9 (1/78)			110.7 (6/54.2)		
8–9.99	21.1 (17/806)			59.0 (39/662)		
10–11.99	7.4 (19/2561)			29.8 (69/2312)		
≥12	7.0 (20/2871)			20.7 (56/2710)		
AIDS diagnosis before initiation						
No	7.5 (39/5176)	1	0.20	24.7 (119/4816)	1	<0.001
Mild ADE (s) only	10.1 (10/993)	0.9 (0.4–1.9)		44.8 (37/826)	1.2 (0.8–1.8)	
Moderate/severe ADE (s)	27.2 (11/404)	1.9 (0.9–4.3)		147.1 (36/245)	3.0 (1.9–4.8)	

cART, combination antiretroviral therapy; ADE, AIDS-defining event; py, person-years; CI, confidence interval.

a Effects were estimated adjusted for *a priori* confounders, time since cART initiation, and current CD4 count, viral load and haemoglobin.

Prior AIDS diagnosis before cART initiation was not predictive of mortality (Table 4). However, among patients not diagnosed with AIDS before starting cART, subsequent diagnosis of mild ADEs and of moderate/severe ADEs after initiation were both associated with mortality, with their impact being similar (rate ratio 4.2 [95% CI 1.9–9.7] and 5.3 [1.8–15.8], respectively; Table 3).

### Associations with Progression to New ADE

Current CD4 count (p<0.001) and haemoglobin (p<0.001) were both strongly prognostic of new ADE, though the magnitude of these associations was weaker compared with that for death (Table 3). Viral load was weakly predictive with increased risk only at levels ≥100,000 copies/ml (p = 0.03), as observed for mortality. Risk of new ADE remained higher during the first 6 months of cART initiation after accounting for current CD4 count, viral load and haemoglobin (p<0.001), but was constant thereafter (p = 0.73).

After adjusting for current CD4 count, viral load and haemoglobin, higher viral load at cART initiation remained strongly associated with new ADE (p<0.001, Table 4), with its effect similar within the first 2 years of initiation compared to after (non-proportional hazards p-value 0.50). CD4 count and haemogolobin at cART initiation had no effect. Patients with moderate/severe ADE (s) before cART initiation had increased risk of new ADE, but not those with mild ADE (s) only (p<0.001).

### Sensitivity Analyses

Results remained the same when either (i) current CD4 count and viral load were defined by assuming a given measurement was valid for a maximum of 6 or 12 months or (ii) patients who initiated on dual therapy before switching to cART were excluded.

## Discussion

This study was based on a well-run, long-term ART programme involving a wide range of public hospitals throughout Thailand, with good quality data collection including accurate ascertainment of AIDS diagnoses and cause of death for most patients. Consistent with other studies from low and high-income settings, CD4 count was found to be the strongest predictor of death and of new ADE [13], [14], [18], [19], [21]. In our cohort, the decline in mortality rate over time from cART initiation (well-documented particularly in low-income settings [6], [13]) was accounted for by current CD4 count, further underlining its key role as an immediate prognostic indicator. As expected, mortality rate at CD4 count <100 cells/mm^3^ was substantially high (124.8 per 100 person-years). Therefore, patients with low CD4 count, even if virologically suppressed, should be closely monitored, with underlying causes of immunosuppression (including potentially non-HIV related conditions) promptly investigated and addressed.

We observed improved survival with increasing CD4 count even at levels above 200 cells/mm^3^, with mortality being rare at ≥500 cells/mm^3^ (rate 1.1 per 1000 person-years). A recent analysis of four large treatment cohorts from Sub-Saharan Africa reported a 1.7 fold increase in mortality risk at CD4 levels 350–499 cells/mm^3^ compared to ≥500 cells/mm^3^ (adjusted for only baseline characteristics and calendar period), comparable to our estimate of 2.5 (95% CI 1.9–3.4) [15]. Furthermore, a French study showed patients on long-term cART with CD4 count >500 cells/mm^3^ had similar mortality rates as the general population [32]. These findings provide growing evidence of the benefit of maintaining CD4 count above 500 cells/mm^3^ while on treatment. They also suggest that relaxing monitoring for patients on treatment with high stable CD4 count should be evaluated for settings with limited capacity for CD4 monitoring. In addition, early HIV diagnosis and early treatment initiation before onset of severe immunosuppression are indicated, given that high CD4 count at ART initiation is associated with improved long-term CD4 cell count recovery [3], [21], [33]; the effect on long-term outcomes of starting ART at CD4 count >500 cells/mm^3^ compared to deferring until CD4 falls below 350 cells/mm^3^ is being investigated in the ongoing randomized START trial [34].

Previous studies across different settings already showed the independent association between low haemoglobin on cART and disease progression [13], [16], [18], [19], [20], [35], as also observed in our study. The link between low haemoglobin and mortality is not well-understood and appears multifactorial, but is likely to be partly due to anaemia being an indicator of other co-morbidities, particularly tuberculosis [35], [36], [37]. Consistent with this, we found 80% of patients dying of tuberculosis had haemoglobin level <10 g/dl at the time of death, compared with 35% of deaths due to other infections and none of those due to non-infection related causes. Although haemoglobin level was lower in women throughout follow-up in our cohort, its prognostic value did not vary by gender (data not shown), consistent with results from a previous UK study [20].

In our cohort, high current viral load was weakly associated with both mortality and new ADE but only at ≥100,000 copies/ml, consistent with a previous ART-CC study [29]. Other studies from low- and high-income settings generally reported a modest association between lack of viral load suppression and poor prognosis [14], [18], [19]. In a large cohort from South Africa, viral load was more strongly prognostic after 12 months, suggesting that viral load monitoring may be more informative after 1 to 2 years of cART [13]. Given the need to prevent accumulation of resistance on a virologically failing regimen, development of viral assays which are cheap, practical and reliable for use in low-income settings remains important for improving patient monitoring [38]. Of interest, high viral load at cART initiation remained predictive of new ADE/death (but not of death) after adjusting for subsequent viral load and CD4 measurements. The explanation for this association, which has not been previously reported, is unclear but could partly be due to delay in diagnosis of certain pre-existing conditions at the time of cART initiation.

Substantial variation in the impact of different ADEs on mortality was previously observed in an ART-CC analysis including treated patients in Europe and US [26]. When applying the prognostic categories of ADEs proposed from this study to our cohort, there was in fact little difference in the effect of mild compared with moderate/severe ADEs on mortality. Our analysis however only included one patient with a severe ADE (progressive multifocal leukoencephalopathy) after starting cART. Furthermore, due to sparse data, we did not evaluate effects of individual ADEs (as was done in the ART-CC analysis), but considered the first event occurring within each ADE prognostic category.

In our study, rate of loss-to follow-up was relatively low, with efforts made to trace patients missing clinic visits using telephone calls and home visits. Although voluntary patient withdrawal from the study was usually due to relocation or ART being accessed elsewhere through the national treatment programs, it is important to note that patients who were either lost to follow-up or voluntarily withdrew were less likely to be virologically suppressed after cART initiation compared to those still in follow-up, with loss to follow-up also associated with lower CD4 attained (data not shown). This could lead to under-estimation of the effects of CD4 count and viral load if patient drop-out was associated with mortality or progression to new ADE’s, conditional on the covariates in the models (including current CD4 count and viral load).

A limitation to our analysis was that effects of covariates were not assessed separately for AIDS-related and non AIDS-related mortality due to limited number of deaths. An association between CD4 count and non-AIDS related mortality has been reported, albeit of weaker magnitude compared with that for AIDS-related mortality [12]. This is consistent with our observation that patients dying of causes not related to infection had higher CD4 count and haemoglobin at the time of death compared to those dying of infection(s), and were also more likely to be virologically suppressed. Of note, we found patients were more likely to die of non-infection related than infection-related causes after 2 years of cART, indicating that ART monitoring potentially needs to account for the changing patterns of mortality over time. Another issue is that most patients with early progression to death and to new ADE were excluded from analyses owing to lack of laboratory measurements after starting cART; risk factors of early mortality in our cohort have been previously described [22]. Consequently, only 14% of new ADEs and 8% of deaths occurred within 6 months of cART initiation, and while immune reconstitution syndrome (IRIS) may account for some of these early events, it is unlikely to play a significant role in our overall findings.

In conclusion, immunosuppression and anaemia may reflect the presence or development of conditions that lead to mortality. Half of the deaths in this Thai cohort were attributable to infections which were chronic and generally considered treatable, while the cause of death remained unknown in nearly a fifth despite regular follow-up. This suggests a significant proportion of patients died of conditions which were not diagnosed in time or appropriately treated, possibly due to constraints in this resource-limited setting and lack of experience in handling such complications at the beginning of the program when the priority was given to starting antiretroviral treatment for all those who urgently needed it. Patients with low CD4 count or haemoglobin should therefore receive more intensive case-management, while monitoring could potentially be relaxed for those with high CD4 if resources are limited. ART monitoring in low-income settings remains a research priority, and in particular, better understanding of predictors of morbidity and mortality due to non-AIDS disease is needed given patients are surviving longer on treatment.
